# Catalytides derived from the Box A region in the ANA/BTG3 protein cleave amyloid-β fragment peptide

**DOI:** 10.1016/j.heliyon.2019.e02454

**Published:** 2019-09-24

**Authors:** Yusuke Hatakawa, Rina Nakamura, Motomi Konishi, Toshiyasu Sakane, Motoaki Saito, Toshifumi Akizawa

**Affiliations:** a Pharmaceutical Technology, Kobe Pharmaceutical University, 4-19-1, Motoyamakita, Higashinada, Kobe, 658-8558, Japan; b O-Force Co., Ltd, 3454 Irino Kuroshio-cho, Hata-gun, Kochi 789-1931, Japan; c Laboratory of Pharmacology, School of Medicine, Kochi University, Kohasu, Oko-cho, Nankoku, Kochi, 783-0047, Japan; d Laboratory of Clinical Analytical Chemistry, Faculty of Pharmaceutical Sciences, Setsunan University, 45-1 Nagaotoge-cho, Hirakata, Osaka 573-0101, Japan

**Keywords:** Biochemistry, Biotechnology, Developmental biology, Molecular biology, Structural biology, Analytical chemistry, Biophysical chemistry, Molecular modeling, Peptides, Chromatography, Catalytide, Hydrolase activity, ANA/BTG3, Tob/BTG, Box A region, Amyloid-β peptide, Alzheimer's disease

## Abstract

We have recently reported about shorter proteolytic peptides termed Catalytide as general name. JAL-TA9 (YKGSGFRMI), a fragment peptide derived from Box A region of Tob1 protein, is the first Catalytide and cleaves Aβ42 and its fragment peptides.

Herein, we demonstrate the enzymatic properties of ANA-TA9 corresponding region to JAL-TA9 in ANA/BTG3 protein. ANA-TA9 showed the auto-proteolytic activity and cleaved 3 kinds of synthetic fragment peptides derived from Aβ42, especially on the central region of Aβ42 with a serine protease like activity. Interestingly, 2 kinds of components, ANA-SA5 (SKGQA) and ANA-YA4 (YRMI), also showed similar proteolytic activity. These results indicate that ANA-TA9 is composed of two different Catalytides.

## 1 Introduction

According to recent reports, Tob/BTG family proteins display antiproliferative activity in a variety of cell types and are involved in the regulation of tumorigenesis [1, 2, 3, 4, 5, 6, 7, 8]. To the best of our knowledge, protease-like activity has never been reported in the Tob/BTG family of proteins or in small synthetic peptides. However, we have previously found the auto-proteolytic activity and the proteolytic activity of the synthetic nona-peptide JAL-TA9 (YKGSGFRMI) derived from the Box A region of the Tob1 protein against Aβ42 and its fragment peptides, Aβ1-20 and Aβ11-29 [9, 10, 11, 12, 13]. Nuclear magnetic resonance (NMR) study proved that the stereo-structure of JAL-TA9 is very compact [14]. Furthermore, we reported the similar proteolytic activity of 5-mer synthetic peptides derived from JAL-TA9 [15]. Therefore, we have termed the shorter proteolytic peptides like JAL-TA9 as Catalytide (Catalytic peptide) [11, 12, 13]. Tob/BTG family proteins are consisted of BTG1, BTG2/Tis21/PC3, ANA/BTG3, BTG4/PC3B, Tob1, and Tob2. Three kinds of homologous regions, Box A, Box B and Box C in the N-terminus region of the Tob/BTG family, are highly conserved among the Tob/BTG family of proteins, but the function of theses region are not clarified (SIFig. 1) [16]. The proteolytic activity of JAL-TA9 suggests that Tob/BTG family proteins may possess the proteolytic activity.

Alzheimer's disease (AD) is the most common age-related neurodegenerative disorder. It is well known that aggregation and accumulation of Aβ42 causes AD due to the strong neurotoxicity of Aβ42 oligomers. This makes Aβ42 an effective target for drug therapies [17, 18, 19, 20, 21, 22, 23, 24, 25, 26]. Mainly, two strategies have been developed against Aβ42. The first utilizes inhibitors against β- or γ-secretases that control the production of soluble Aβ42 [22]. The other uses inhibitors of Aβ42 oligomerization [17]. Many trials have been conducted to develop drugs for the treatment of AD, but the results have not been encouraging [25, 26, 27, 28, 29, 30]. Thus, the development of new and effective drugs is an urgent necessity for treating AD. Catalytides such as JAL-TA9 are attractive candidates as peptide drugs with a novel strategy for prevention and treatment of AD.

In this study, we focus on ANA-TA9 (SKGQAYRMI) derived from Box A region of the ANA/BTG3 protein corresponding to JAL-TA9 to find new Catalytide. Herein, we demonstrate the proteolytic activity of ANA-TA9 and its components, ANA-SA5 (SKGQA) and -YA4 (YRMI).

## 2 Materials and methods

### 2.1 Chemical synthesis of peptides

Peptides were synthesized from Fmoc-protected L-amino acid derivatives according to the method described by Kojima *et al.* [31] by using an automated peptide synthesizer (model 433A, Applied Biosystems, California, U. S. A., 0.1 mmol scale with preloaded resin). After deprotection according to the manufacturer's protocol, each peptide was purified using a reversed-phase HPLC (Capcell Pak C18 column, SG, 10 or 15 mm i.d. × 250 mm; Shiseido Co., Ltd., Japan) with a linear elution gradient from 0.1 % trifluoro acetic acid (TFA) to 50 % or 70 % CH_3_CN containing 0.1 % TFA over 30 min. The flow rate was set at 3 or 6 mL/min. The primary peak fractions were collected and then lyophilized. The purity of the synthetic peptides and the progress of the enzymatic reaction were confirmed by an analytical reversed-phase HPLC (Capcell Pak C18 column, MGII, 4.6 mm i.d. × 150 mm; Shiseido Co., Ltd., Japan) at a flow rate of 1.0 mL/min with a linear elution gradient from 0.1 % TFA to 70 % CH_3_CN containing 0.1 % TFA. The column eluate was monitored with a photodiode-array detector (SPD-M20A; Shimadzu, Japan). Each purified peptide was characterized by ESI-MS using a Qstar Elite Hybrid LC-MS/MS system.

### 2.2 Analysis of proteolytic activity and determination of cleavage sites

ANA-TA9, -SA5 and -YA4 (final conc., 0.2 mM) were individually incubated with or without the Aβ42 fragment peptides (Aβ1-20, Aβ11-29 or Aβ28-42) in the presence of human serum albumin (HSA) (final conc., 0.025 % w/v) in PBS (pH 7.4) at 37 °C. A portion of the reaction mixture was analyzed in a time-dependent manner on the analytical HPLC system described above. The peak fractions monitored at 220 nm were collected into microtubes (Eppendorf Safe-Lock Tubes, 1.5 mL).

After lyophilization, the appropriate quantity of 36 % CH_3_CN containing 0.1 % HCOOH was determined based on the chromatographic peak height and added with stirring by an automatic mixer. The cleavage sites were determined by ESI-MS using the flow injection method with 70 % CH_3_CN containing 0.1 % HCOOH on a Qstar Hybrid LC-MS/MS system (ABI). The flow rate was set at 0.1 mL/min.

### 2.3 Kinetic parameters

The enzymatic activity of ANA-TA9, -SA5 and -YA4 were measured using Aβ11-29 as substrates. ANA-TA9, -SA5 and -YA4 were incubated with Aβ11-29 in the same manner described in analysis of proteolytic activity. The reaction mixture (10 μL) was analyzed on an analytical HPLC at the initial time and after 24 hrs of incubation. The peak heights of Aβ11-29 gave linear fits on the Lineweaver-Burk plots.

### 2.4 Structural analysis by the computer modeling

The computer modeling was performed using the Software CSC Chem 3D UltraTM ver. 9.0. At first, all peptide bond angles and dihedral angles fix to 180°. Second, the six atoms organizing a peptide bond were arranged in one plane, and then settled the bond length. After settled these, we carried out calculation by the structural optimization and energy minimization according to MM2 and MMFF94 parameters (bond length, bond angles, torsion, dipole-moment, and van der Waals values).

## 3 Results

### 3.1 Preparation of synthetic peptides

Three kinds fragment peptides derived from the Box A region of the ANA/BTG3 protein and 3 kinds of Aβ-fragment peptides were synthesized. All peptides were purified using a reversed-phase preparative high-performance liquid chromatography (HPLC). The purity of each peptide was checked using an analytical HPLC system with a photo-diode array detector and NMR. The molecular weights of purified peptides were confirmed using Mass Spectrometry (MS) (Table 1). On the basis of these data, we concluded that all peptides were adequately purified to perform the following experiments (SIFigs. 2 and 3).

**Table 1:** Purity confirmation and identification of each peptide. Amino acid sequence and identification of each molecular weight by MS analysis.

Peptide	Sequence	Theoretical MS	Experimental MS
ANA-TA9	SKGQAYRMI	1052.54	1052.5680
ANA-SA5	SKGQA	489.25	489.2685
ANA-YA4	YRMI	581.30	581.3164
Aβ1-20	DAEFRHDSGYEVHHQKLVFF	2460.16	2460.3244
Aβ11-29	EVHHQKLVFFAEDVGSNKG	2140.07	2140.1220
Aβ28-42	KGAIIGLMVGGVVIA	1396.85	1396.8604

### 3.2 The auto-proteolytic activity

**Fig. 1:**
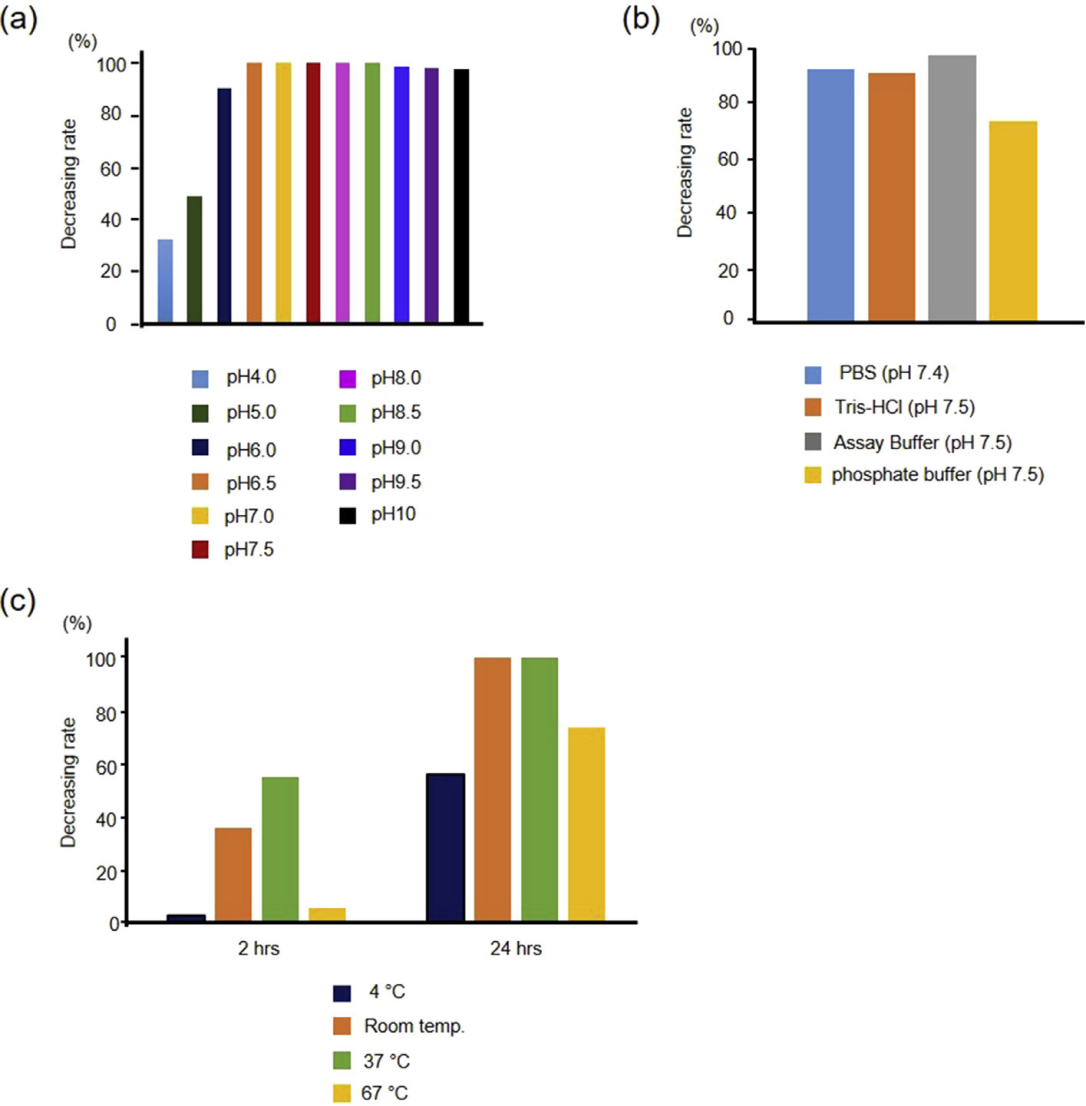
Determination of the optimal conditions for the auto-proteolytic activity of ANA-TA9. The auto-proteolytic activity was examined in the presence of HSA. (a) pH dependence. The optimal pH was examined in 50 mM Tris-HCl, and ANA-TA9 showed high activity above pH 6.5. (b) Effects of various buffers. PBS, Tris-HCl and assay buffer (50 mM Tris-HCl (pH 7.5), 150 mM NaCl, 10 mM Ca^2+^, 5 μM Zn^2+^, 0.06 % Bril35 0.02 % NaN_3_) [32], which was higher than that observed in Phosphate buffer. (c) Temperature. The optimal temperature was examined in PBS (pH 7.4), and ANA-TA9 showed the highest auto-proteolytic activity at 37 °C.

We first examined pH dependency, which involved studying the effects of various reaction buffers and reaction temperatures on the proteolytic activity of ANA-TA9. To determine the optimal reaction conditions for measuring the auto-proteolytic activity, we tested the peptides in the presence of human serum albumin (HSA) using an analytical HPLC. The pH dependency test was performed between the pH range of 4.0 and 10.0 in Tris-HCl buffer. Optimal proteolytic activity was observed at pH 6.5 and remained stable thereafter (Fig. 1a). The activity was unaffected by different buffers such as PBS (pH 7.4), Tris-HCl (pH 7.5) and Assay Buffer (pH 7.5); however, decreased activity was observed in Phosphate Buffer (PB) (Fig. 1b). The optimal temperature for proteolytic activity was found to be 37 °C (Fig. 1c). Thus, it was decided to examine the proteolytic activity of ANA-TA9 in phosphate buffer saline (PBS) (pH 7.4) with HSA at 37 °C, since these reaction conditions are similar to physiological conditions.

**Fig. 2:**
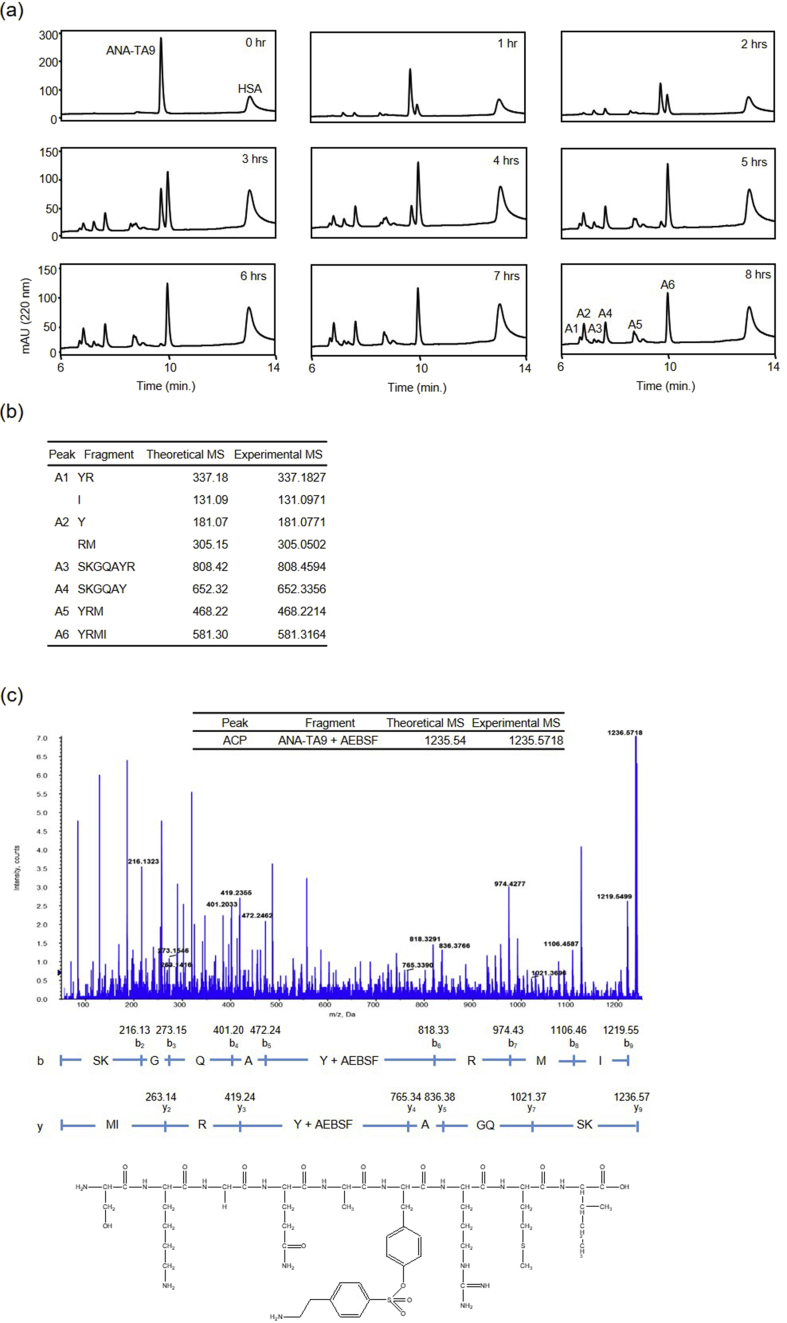
The auto-proteolytic activity of ANA-TA9 and MS/MS analysis. ANA-TA9 (final conc., 0.2 mM) was incubated in the presence of HSA (final conc., 0.025 % w/v) in PBS at 37 °C. (a) Ten μL of the reaction mixture at every 1 hr for 8 hrs was analyzed on an analytical HPLC. (b) The cleavage sites were determined by ESI-MS using a flow injection method on a Qstar Hybrid LC-MS/MS system (ABI). (c) MS/MS analysis of the complex with ANA-TA9 and AEBSF.

**Fig. 3:**
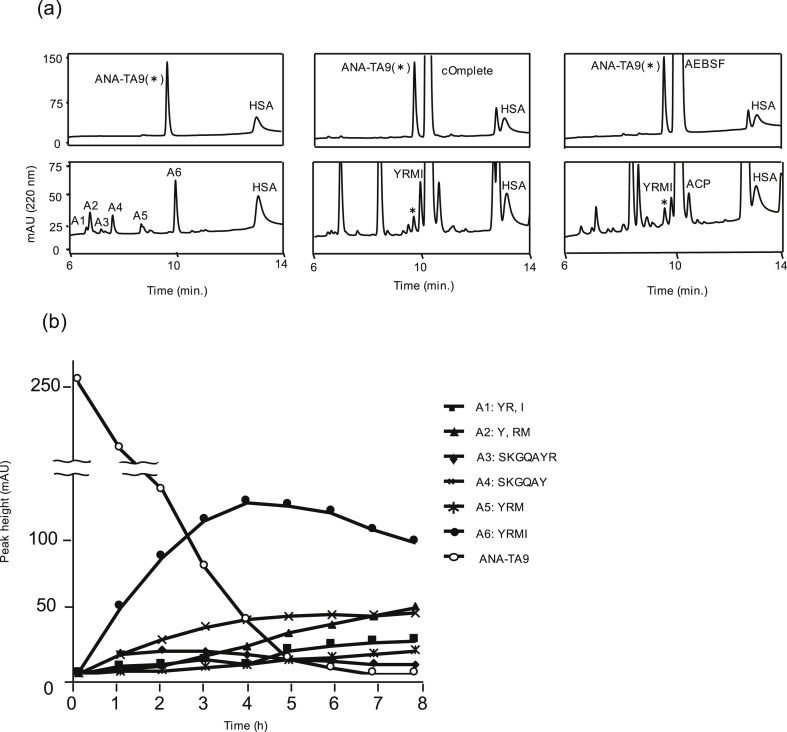
The auto-proteolytic activity of ANA-TA9. (a) ANA-TA9 (final conc.: 0.2 mM) was incubated in presence of human serum albumin (HSA) (final conc.: 0.025 % w/v) in PBS at 37 °C without (a left) or with protease inhibitors (center and right). 10 μL of the reaction mixture at 0 hr (upper) or 6 hrs (lower) were analyzed by an analytical HPLC. The determination of cleavage site was performed by ESI-MS. A1 to A6 were identified as fragment peptide of ANA-TA9. The peak ACP was determined as the complex with ANA-TA9 and AEBSF was determined (a right). (b) Peak plot of each fragment peptides produced from ANA-TA9 by time dependent analysis.

The auto-proteolytic activity was monitored hourly for 8 hrs (Fig. 2a). ANA-TA9 was the only peptide detected as a single peak in the initial stages of the reaction; however, several new peaks were observed after 1 hr. The increase in peak heights was proportional to decrease in concentration of ANA-TA9 for up to 5 hrs. ANA-TA9 was almost untraceable after 5 hrs. Six peaks were identified after a period of 6 hrs. We determined their amino acid sequence by MS. As a result, 6 kinds of peptide fragments derived from ANA-TA9 and 2 amino acids were identified, but no fragments produced from HSA were identified (Figs. 2a, b and 3a left). Similar results were obtained from the reaction mixture after 8 hrs (Fig. 2a). These results demonstrate the proteolytic activity of ANA-TA9; however, HSA remained unaffected by it. A total of 2 peptides, ANA-TA9 and YRMI, were identified in the presence of protease inhibitor cocktail (cOmplete) (Fig. 3a center) or 4-(2-aminoethyl) benzenesulfonyl fluoride hydrochloride (AEBSF) (Fig. 3a right). The peak corresponding to ACP (AEBSF Coupled Peptide) was identified within the complex of ANA-TA9 and AEBSF. The binding site of AEBSF was identified as a Tyr residue through MS/MS analysis (Fig. 2c). No other peptide peaks were observed. These results indicate that the auto-proteolytic activity of ANA-TA9 was partially inhibited by AEBSF.

Next, we plotted the peak heights of newly appearing peptide fragments on the chromatogram. YRMI showed a steady increase in proportion to the decrease of ANA-TA9 for 4 hrs (Fig. 3b). A6 (YRMI) showed a steady decrease and A2 (Y and RM) showed a steady increase after the complete degradation of ANA-TA9 at 6 hrs. These data suggest that YRMI may exhibit the auto-proteolytic activity similar to ANA-TA9. Accordingly, we examined the proteolytic activity of ANA-TA9 and its fragments, YRMI and SKGQA.

### 3.3 The proteolytic activity of ANA-TA9 and its fragments against Aβ42 fragments

**Fig. 4:**
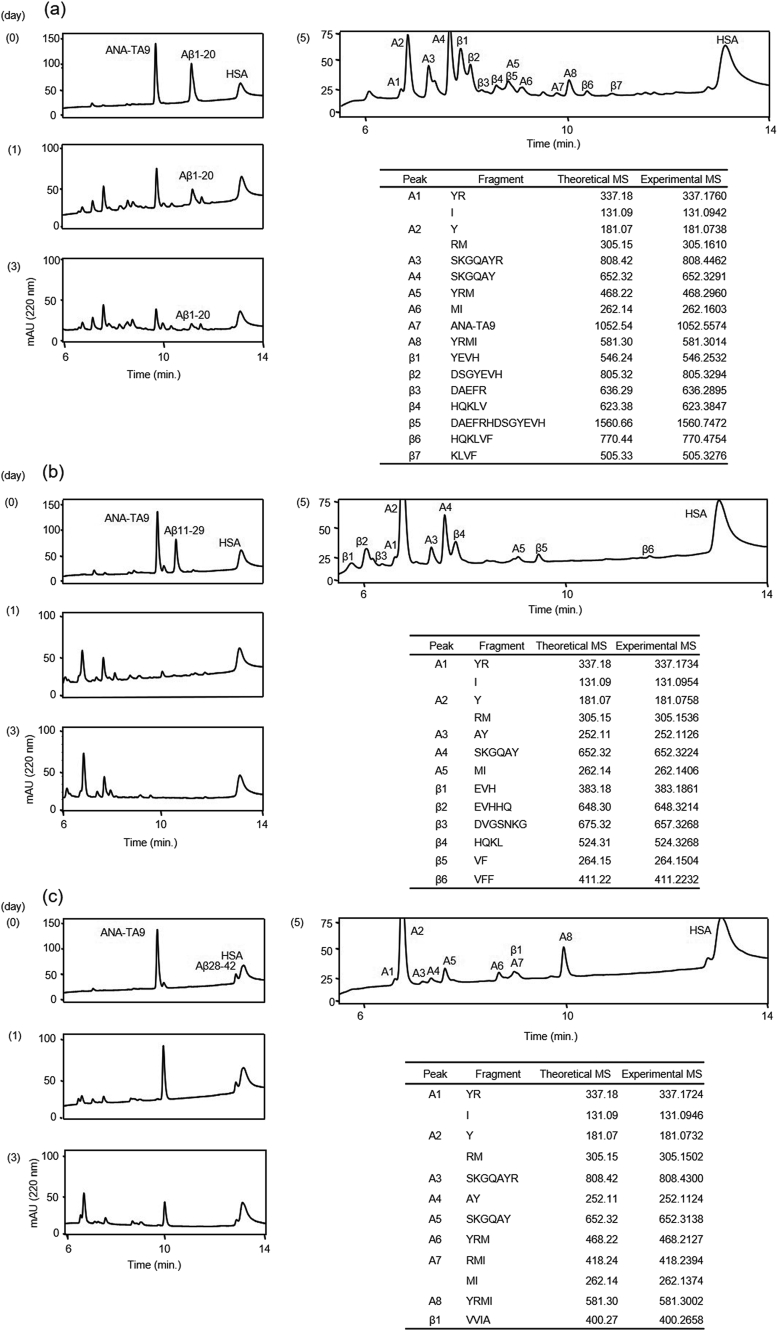
Cleavage reaction of Aβ fragment peptides by ANA-TA9. Time dependent analysis of the reaction mixture of ANA-TA9 and Aβ-Fs on 0, 1 and 3 days. On 5 days each peak were corrected and applied MS analysis to determine the cleavage site. (a) Aβ1-20 (b) Aβ11-29 (c) Aβ28-42.

We investigated the proteolytic activity of ANA-TA9 on 3 kinds of Aβ-derived fragment peptide, Aβ1-20, Aβ11-29 and Aβ28-42 [32]. The reaction mixture was analyzed by an analytical HPLC daily for 5 days (Fig. 4). The reaction containing Aβ1-20 showed a gradual and timely decrease of ANA-TA9. The chromatograms also showed gradual changes from day 1 to day 5 (Fig. 4a). The peptide Aβ1-20 distinguishably decreased on day 1 and was almost untraceable on day 3. The resulting peaks were analyzed on MS at day 5. We were able to identify 7 kinds of peptides (β1-7) were identified as peptide fragments from Aβ1-20 (Fig. 4a). In the reaction containing Aβ11-29, 6 kinds of peptide (β1-6) were identified as peptide fragments from Aβ11-29 (Fig. 4b). The alternating peak heights of each peptide fragment indicated that Aβ11-29 was almost completely cleaved on day 1. Interestingly, although ANA-TA9 was almost untraceable on day 1, the chromatogram patterns showed variations until day 5 (Fig. 4b). It was also observed that Aβ28-42 showed weak resistance to ANA-TA9 in comparison with Aβ1-20 and Aβ11-29. Only ^39^VVIA^42^ was identified as a peptide fragment from Aβ28-42 (Fig. 4c). Taken together, these results indicate that ANA-TA9 has higher activity against Aβ1-20 and Aβ11-29 than against Aβ28-42.

**Fig. 5:**
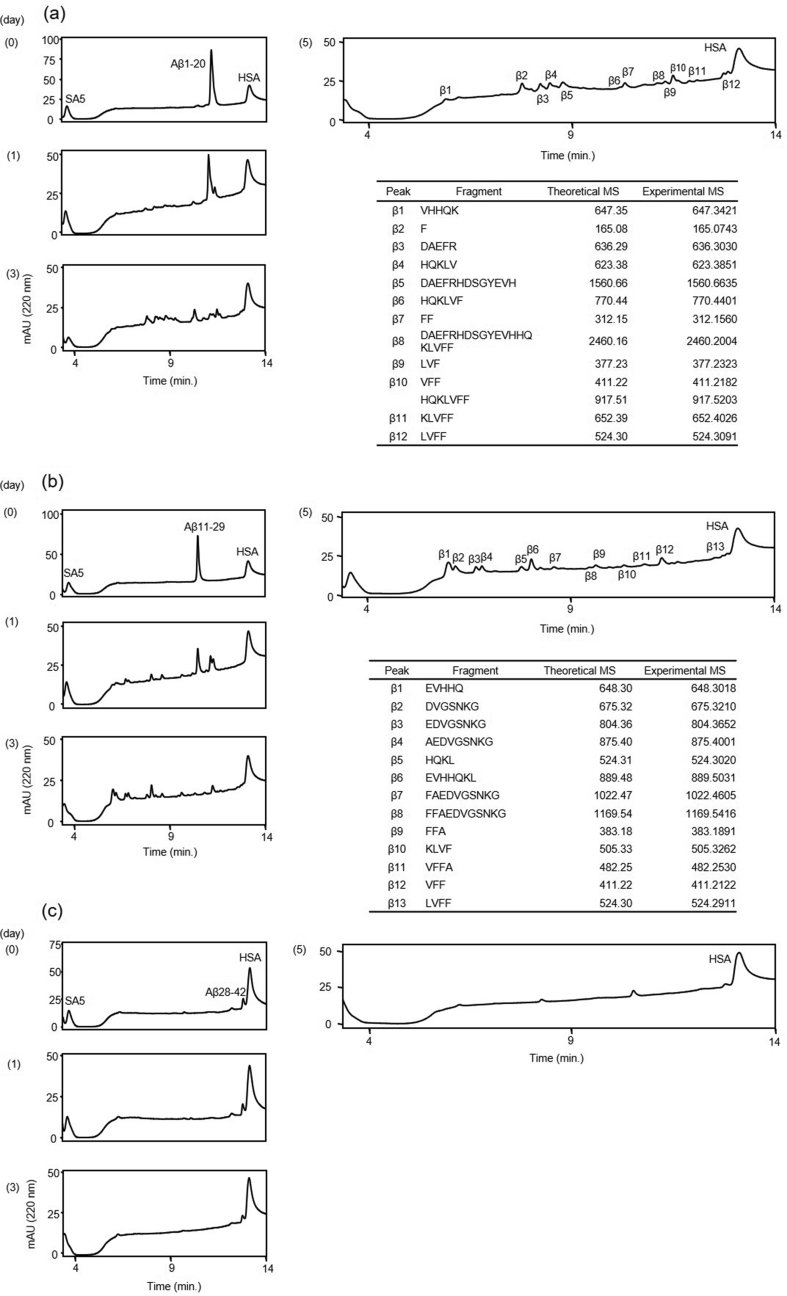
Cleavage reaction of Aβ fragment peptides by ANA-SA5. Time dependent analysis of the reaction mixture of ANA-SA5 and Aβ-Fs on 0, 1 and 3 days. On 5 days each peak were corrected and applied MS analysis to determine the cleavage site. (a) Aβ1-20 (b) Aβ11-29 (c) Aβ28-42.

**Fig. 6:**
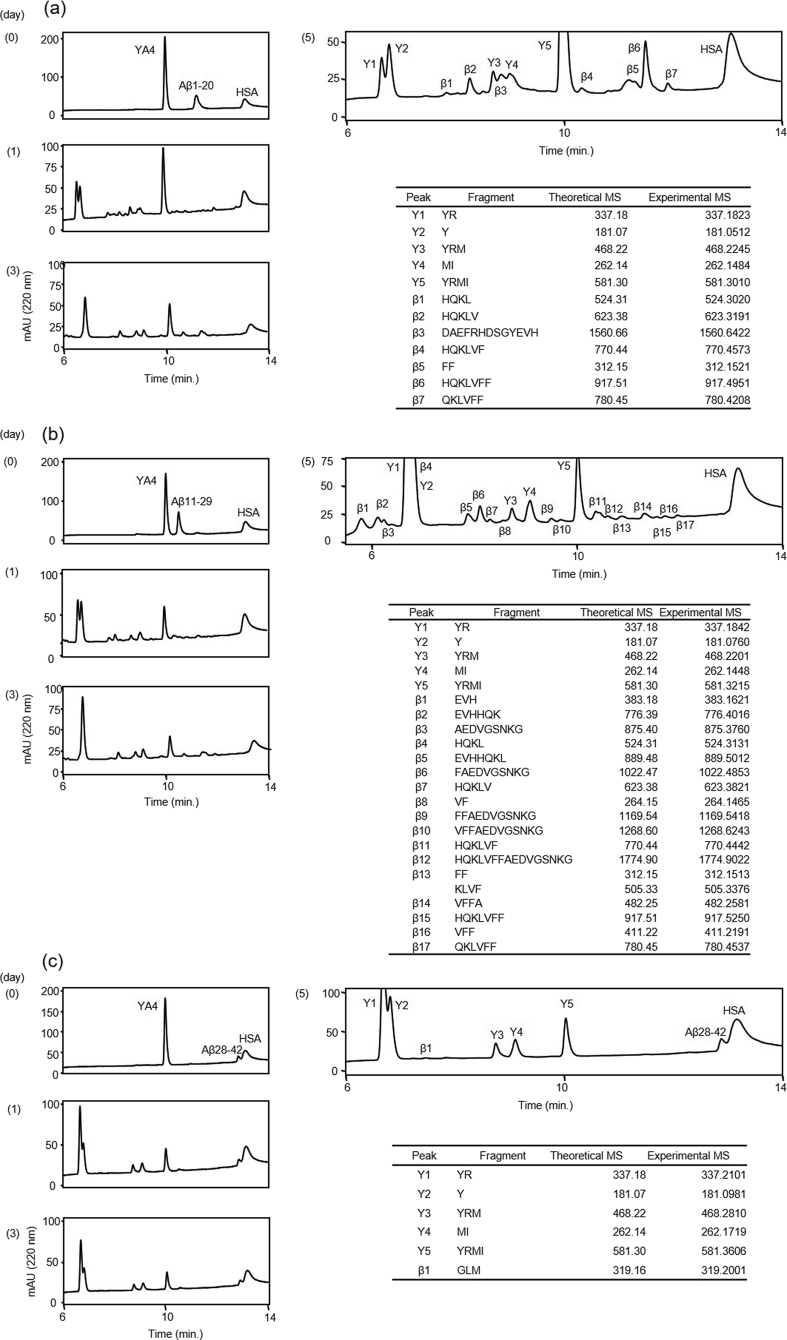
Cleavage reaction of Aβ fragment peptides by ANA-YA4. Time dependent analysis of the reaction mixture of ANA-YA4 and Aβ-Fs on 0, 1 and 3 days. On 5 days each peak were corrected and applied MS analysis to determine the cleavage site. (a) Aβ1-20 (b) Aβ11-29 (c) Aβ28-42.

The proteolytic activities of both ANA-SA5 and ANA-YA4 were also investigated in a manner similar to ANA-TA9 (Figs. 5 and 6). In the case of ANA-SA5, we identified 12 derivative peptide fragments from Aβ1-20 and 13 peptide fragments from Aβ11-29 (Figs. 5a and b). No fragments were observed in the Aβ28-42 reaction (Fig. 5c). The reaction with ANA-YA4, revealed 7 fragments from Aβ1-20 and 17 fragments from Aβ11-29 (Figs. 6a and b). Only 1 fragment from Aβ28-42 was identified (Fig. 6c).

**Fig. 7:**
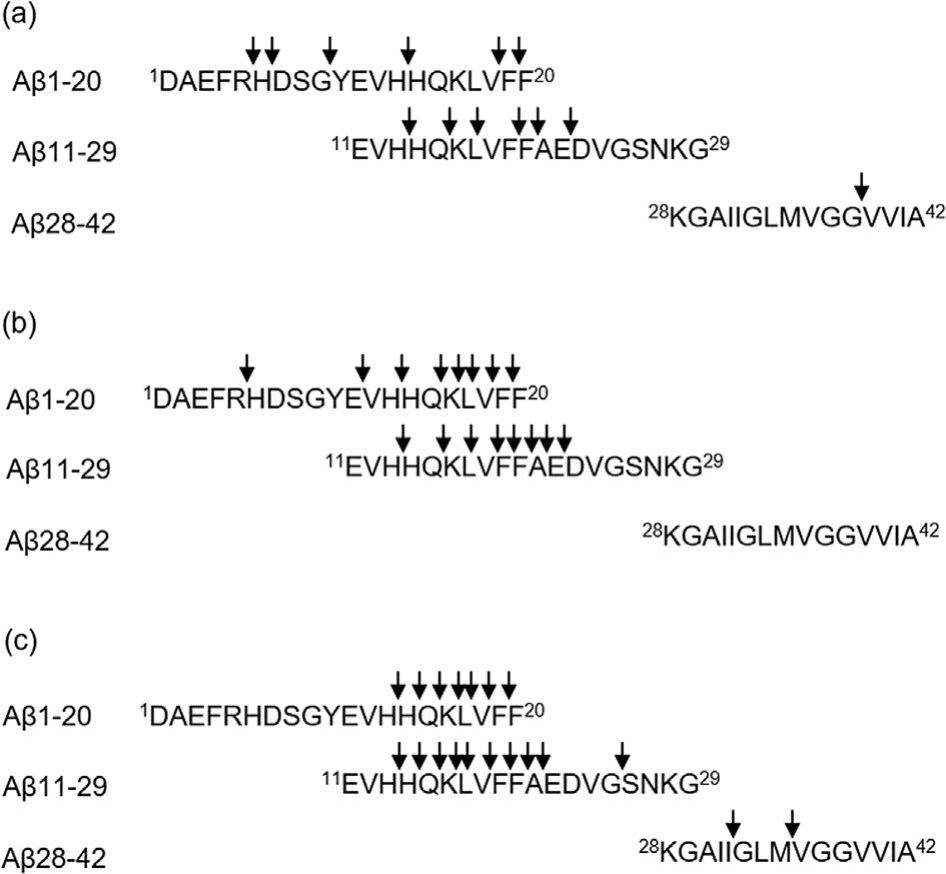
Cleavage sites on Aβ42 and its fragment peptides. (a) ANA-TA9 (b) ANA-SA5 (c) ANA-YA4.

The cleavage sites of the fragments were determined through MS analyses (Fig. 7). The results indicate that all peptides cleaved the Aβ-fragment peptides, especially the central region of Aβ42 similar to JAL-TA9 [11, 12, 13]. Therefore, the next step was to calculate the kinetic parameters of Aβ11-29 which is known to form β-sheets and contain the regions essential for oligomerization/aggregation of Aβ42 [33].

### 3.4 Kinetic parameters

**Fig. 8:**
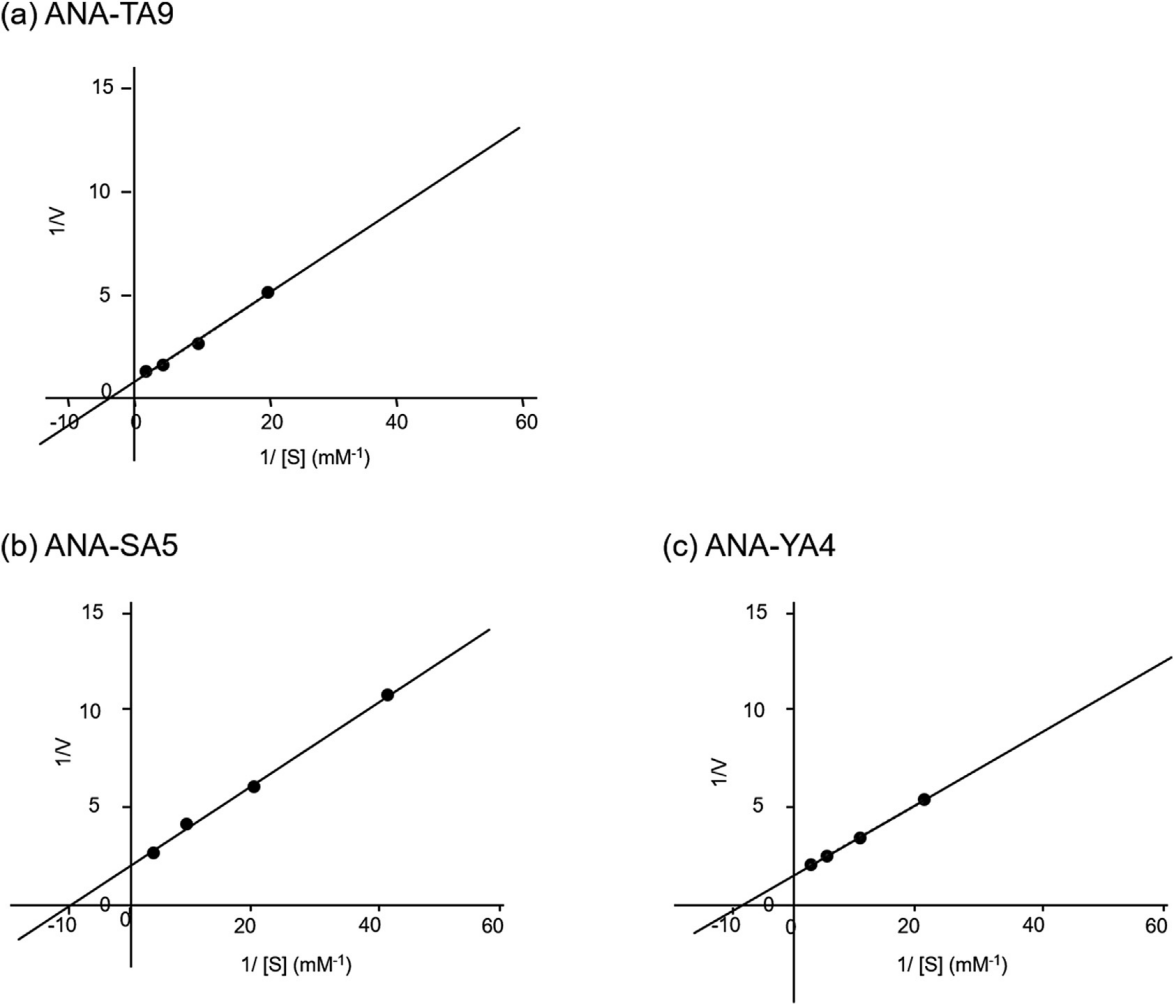
Lineweaver-Burk plot. (a) ANA-TA9 (b) ANA-SA5 (c) ANA-YA4.

The *K*_m_, *V*_max_ and *k*_cat_ values of the cleavage reactions of Aβ11-29 by ANA-TA9 were 0.32 mM, 1.47 nmol/hr and 1.23 × 10^−3^/min, those by ANA-SA5 were 0.13 mM 0.57 nmol/hr and 4.75 × 10^−4^/min, and those by ANA-YA4 were 0.15 mM, 0.80 nmol/hr and 6.67 × 10^−4^/min, respectively (Fig. 8 and SIFig. 4).

## 4 Discussion

The biological substrates of ANA-TA9 are still not known. However, this study has demonstrated that ANA-TA9 consists of 2 different catalytic peptides, ANA-SA5 and -YA4. Considering together with the unstability of the Tob/BTG protein [34], ANA/BTG3 protein may have proteolytic activity.

Furthermore*,* it is important to consider the side effects of ANA-TA9 before its clinical use. For this, we examined the proteolytic activity of ANA-TA9 against 4 native proteins, γ-globulin, rabbit immuno-globulin G, cytochrome C, and lysozyme. The data obtained from HPLC proved that ANA-TA9 has no activity against these 4 native proteins or against HSA (SIFig. 5). This suggests that ANA-TA9 is an attractive candidate for development of a novel peptide drug that could be applicable in clinical settings for prevention and treatment of AD without any serious side effects.

**Fig. 9:**
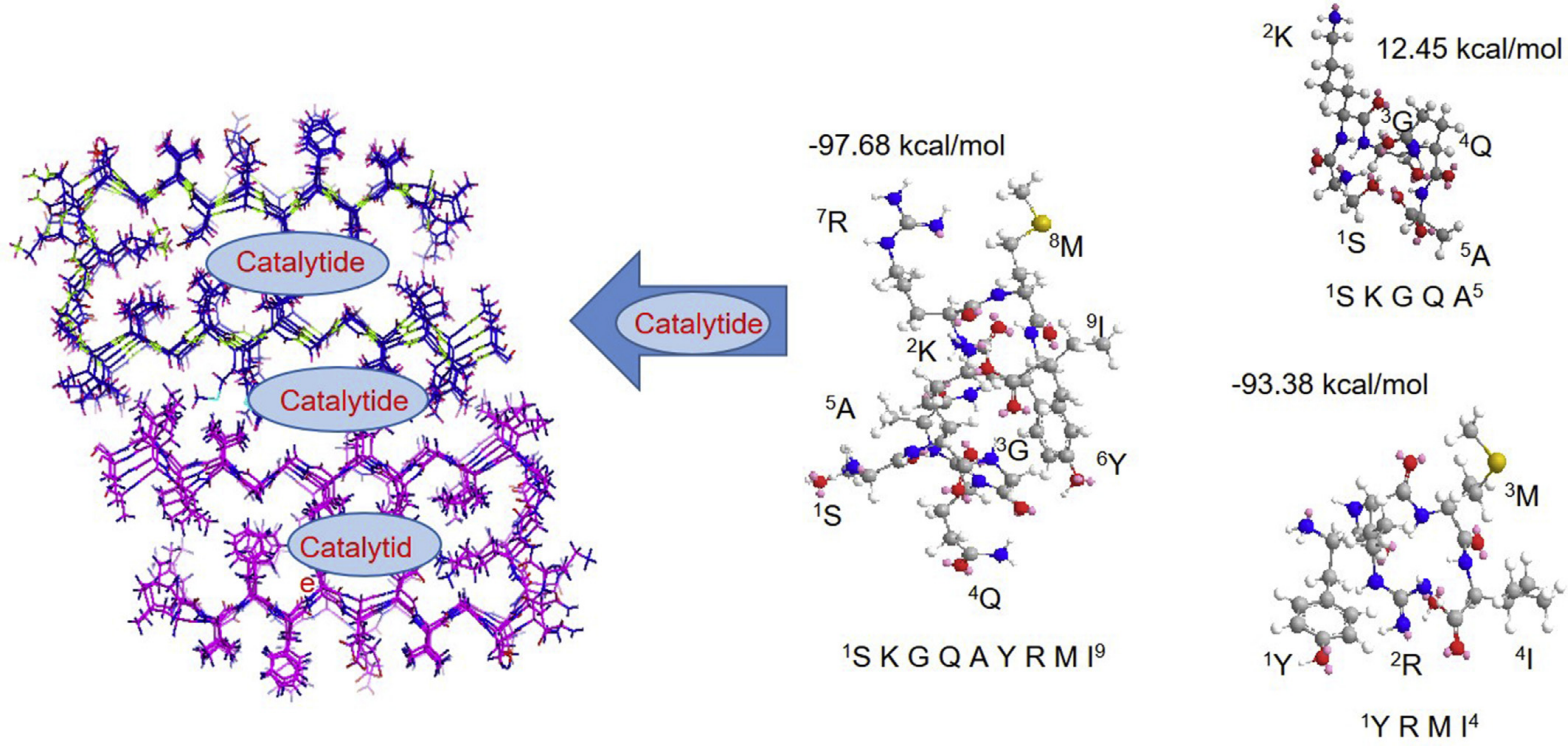
Estimated cleavage mechanism of oligomeraized/aggregated Aβ42 by ANA-TA9, -SA5 and -YA4. The stereo-structure was estimated by using Chem 3D soft wear (right). After then, the steric energy was calculated by using MM2 and MMFF94 (right). The estimated structure of ANA-TA9 formed the compact structure. Thus, ANA-TA9, -SA5 and -YA4 can enter the aggregated/oligomerized Aβ42 (left) and cleaved.

The final question was why Catalytides cleave Aβ42 derived fragment peptides (Aβ-Fs) especially Aβ11-29 which is important in the aggregated/oligomerized Aβ42 [35], in spite of its short sequence and lack of binding. Conformational analyses carried out using the computer modeling platforms MM2 and MMFF94 (SIFig. 6) [12,14] had shown that ANA-TA9, -SA5 and -YA4 form compact structures (Fig. 9). The molecular sizes of these peptides were similar to that of JAL-TA9 [11, 12, 13, 14, 15]. The complete mechanism remains to be elucidated; however, we would like to present a hypothesis to explain the cleavage of Aβ-Fs by Catalytides. We hypothesize that the smaller Catalytides form a compact structure invading the inner space of the aggregated/oligomerized Aβ-Fs and remain in the region without binding site to the substrate (Fig. 9). As a result, the aggregated/oligomeraized proteins is cleaved from inside.

We conclude that 3 kinds of the synthetic peptides, ANA-TA9, -SA5, and -YA4, were the Catalytide derived from the Box A region of the ANA/BTG3 protein. These fragments cleave Aβ42 peptide fragments through proteolysis in a manner similar to JAL-TA9. In addition, ANA/BTG3 protein is also predicted to have hydrolase activity.

The findings presented here are novel, as similar studies have not been attempted so far, to the best of our knowledge. These findings are expected to have applications in various aspects of medical and academic pursuits as well as in the study of the mechanism of proteolysis.

## Declarations

### Author contribution statement

Yusuke Hatakawa: Performed the experiments; Analyzed and interpreted the data; Contributed reagents, materials, analysis tools or data.

Rina Nakamura: Performed the experiments; Analyzed and interpreted the data; Contributed reagents, materials, analysis tools or data; Wrote the paper.

Motomi Konishi: Performed the experiments; Analyzed and interpreted the data.

Toshiyasu Sakane, Motoaki Saito: Conceived and designed the experiments; Wrote the paper.

Toshifumi Akizawa: Conceived and designed the experiments; Analyzed and interpreted the data; Contributed reagents, materials, analysis tools or data; Wrote the paper.

### Funding statement

This work was supported by the Japan Society for the Promotion of Science (JSPS) Grants-in-Aid for Scientific Research (KAKENHI) Program, Grant Number 15K07908, and the Okinawa Institute of Science and Technology (OIST) Proof-of Concept (POC) Program.

### Competing interest statement

The authors declare no conflict of interest.

### Additional information

No additional information is available for this paper.

## Appendix A Supplementary data

ANA-1 SFig ver.3 final YH.pptx ANA-1 SFig ver.3 final YH.pptx
