# Supplementary material for: Catalytides derived from the Box A region in the ANA/BTG3 protein cleave amyloid-β fragment peptide
Source: Heliyon. 2019 Sep 24;5(9):e02454. doi: 10.1016/j.heliyon.2019.e02454 (PMC6819762; doi:10.1016/j.heliyon.2019.e02454)
Supplement: ANA-1 SFig ver.3 final YH.pptx [file mmc1.pptx]

## Slide 1
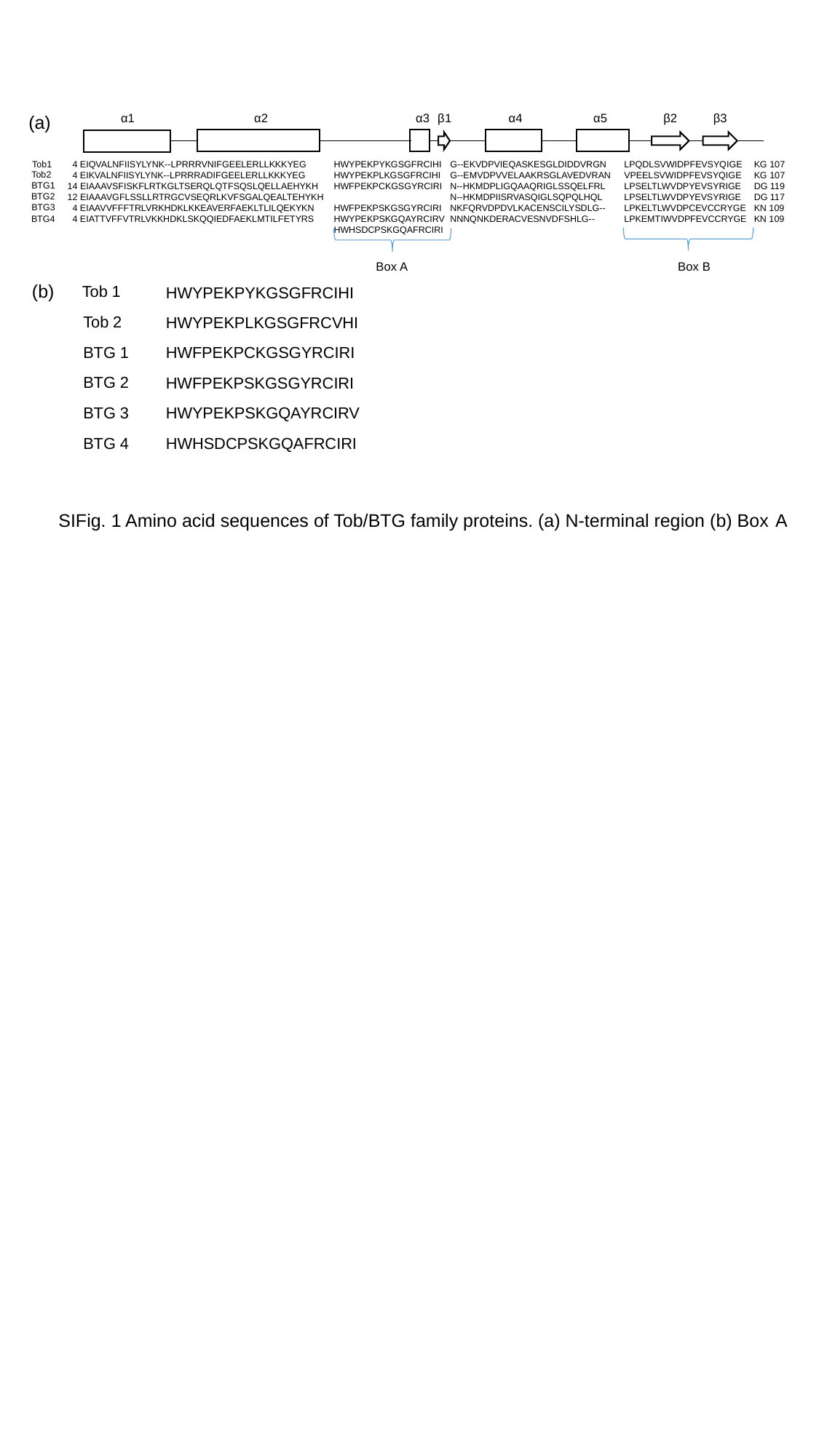

α1
α2
α3
β1
α4
α5
β2
β3
(a)
Tob1
 4 EIQVALNFIISYLYNK--LPRRRVNIFGEELERLLKKKYEG
 4 EIKVALNFIISYLYNK--LPRRRADIFGEELERLLKKKYEG
14 EIAAAVSFISKFLRTKGLTSERQLQTFSQSLQELLAEHYKH
12 EIAAAVGFLSSLLRTRGCVSEQRLKVFSGALQEALTEHYKH
 4 EIAAVVFFFTRLVRKHDKLKKEAVERFAEKLTLILQEKYKN
 4 EIATTVFFVTRLVKKHDKLSKQQIEDFAEKLMTILFETYRS
HWYPEKPYKGSGFRCIHI
HWYPEKPLKGSGFRCIHI
HWFPEKPCKGSGYRCIRI　HWFPEKPSKGSGYRCIRI
HWYPEKPSKGQAYRCIRV
HWHSDCPSKGQAFRCIRI
G--EKVDPVIEQASKESGLDIDDVRGN
G--EMVDPVVELAAKRSGLAVEDVRAN
N--HKMDPLIGQAAQRIGLSSQELFRL
N--HKMDPIISRVASQIGLSQPQLHQL
NKFQRVDPDVLKACENSCILYSDLG--
NNNQNKDERACVESNVDFSHLG--
LPQDLSVWIDPFEVSYQIGE
VPEELSVWIDPFEVSYQIGE
LPSELTLWVDPYEVSYRIGE
LPSELTLWVDPYEVSYRIGE
LPKELTLWVDPCEVCCRYGE
LPKEMTIWVDPFEVCCRYGE
KG 107
KG 107
DG 119
DG 117
KN 109
KN 109
Tob2
BTG1
BTG2
BTG3
BTG4
Box A
Box B
(b)
Tob 1
HWYPEKPYKGSGFRCIHI
Tob 2
HWYPEKPLKGSGFRCVHI
BTG 1
HWFPEKPCKGSGYRCIRI
BTG 2
HWFPEKPSKGSGYRCIRI
BTG 3
HWYPEKPSKGQAYRCIRV
BTG 4
HWHSDCPSKGQAFRCIRI
SIFig. 1 Amino acid sequences of Tob/BTG family proteins. (a) N-terminal region (b) Box A

## Slide 2
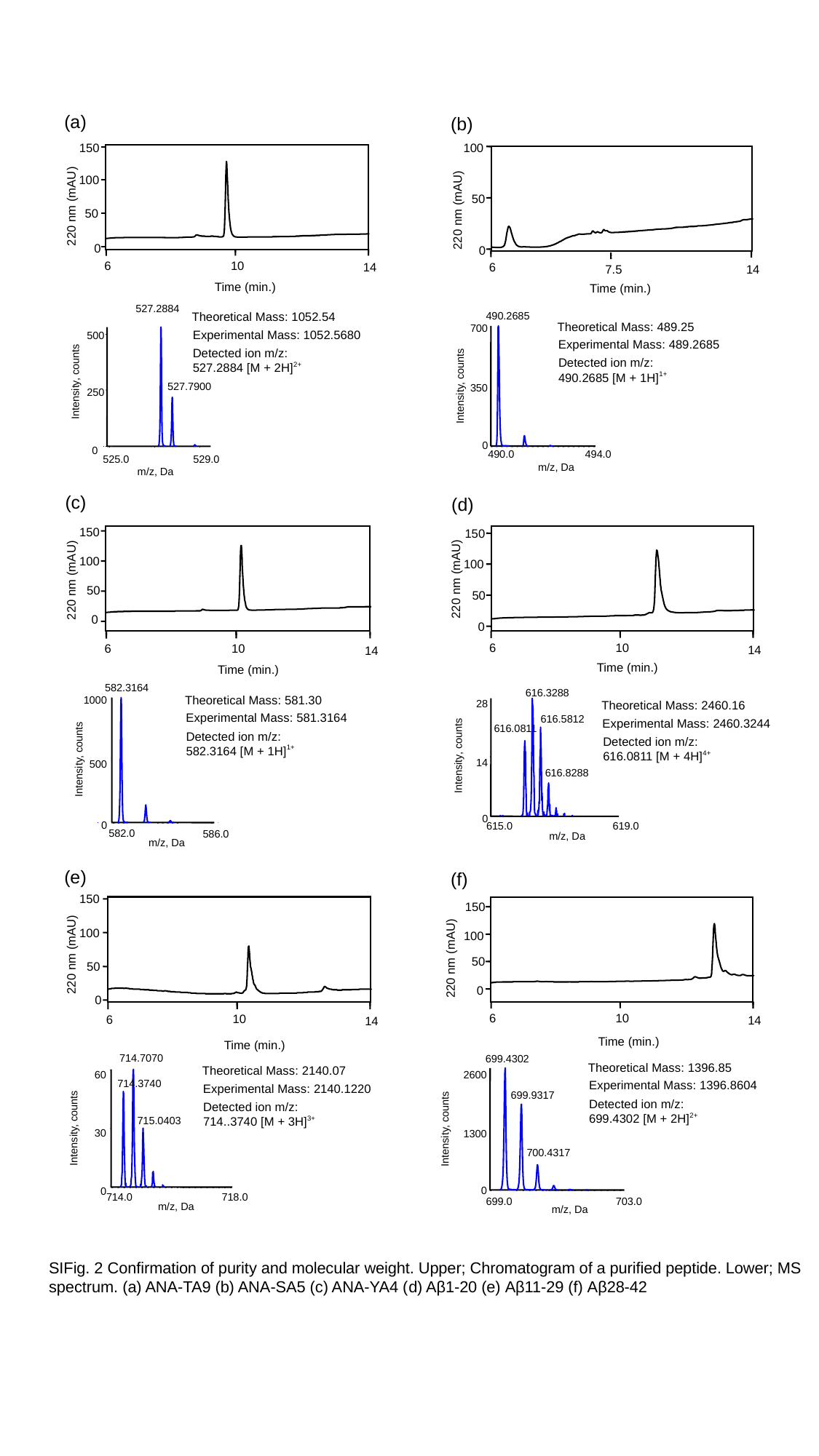

(a)
(b)
150
100
220 nm (mAU)
50
0
10
6
14
Time (min.)
100
220 nm (mAU)
50
0
6
7.5
14
Time (min.)
527.2884
500
Intensity, counts
527.7900
250
0
525.0
529.0
Theoretical Mass: 1052.54
Experimental Mass: 1052.5680
Detected ion m/z:
527.2884 [M + 2H]2+
m/z, Da
490.2685
Theoretical Mass: 489.25
700
Experimental Mass: 489.2685
Detected ion m/z:
490.2685 [M + 1H]1+
Intensity, counts
350
0
490.0
494.0
m/z, Da
(c)
(d)
150
220 nm (mAU)
100
50
0
10
6
14
Time (min.)
150
100
220 nm (mAU)
50
0
10
6
14
Time (min.)
582.3164
Theoretical Mass: 581.30
1000
Experimental Mass: 581.3164
Detected ion m/z:
582.3164 [M + 1H]1+
Intensity, counts
500
0
582.0
586.0
m/z, Da
616.3288
Theoretical Mass: 2460.16
28
Experimental Mass: 2460.3244
616.5812
616.0811
Detected ion m/z:
616.0811 [M + 4H]4+
Intensity, counts
14
616.8288
0
615.0
619.0
m/z, Da
(e)
(f)
150
100
220 nm (mAU)
50
0
10
6
14
Time (min.)
150
100
50
0
220 nm (mAU)
10
6
14
Time (min.)
714.7070
Theoretical Mass: 2140.07
60
Experimental Mass: 2140.1220
714.3740
Detected ion m/z:
714..3740 [M + 3H]3+
715.0403
Intensity, counts
30
0
714.0
718.0
m/z, Da
699.4302
Theoretical Mass: 1396.85
2600
Experimental Mass: 1396.8604
699.9317
Detected ion m/z:
699.4302 [M + 2H]2+
Intensity, counts
1300
700.4317
0
699.0
703.0
m/z, Da
SIFig. 2 Confirmation of purity and molecular weight. Upper; Chromatogram of a purified peptide. Lower; MS spectrum. (a) ANA-TA9 (b) ANA-SA5 (c) ANA-YA4 (d) Aβ1-20 (e) Aβ11-29 (f) Aβ28-42

## Slide 3
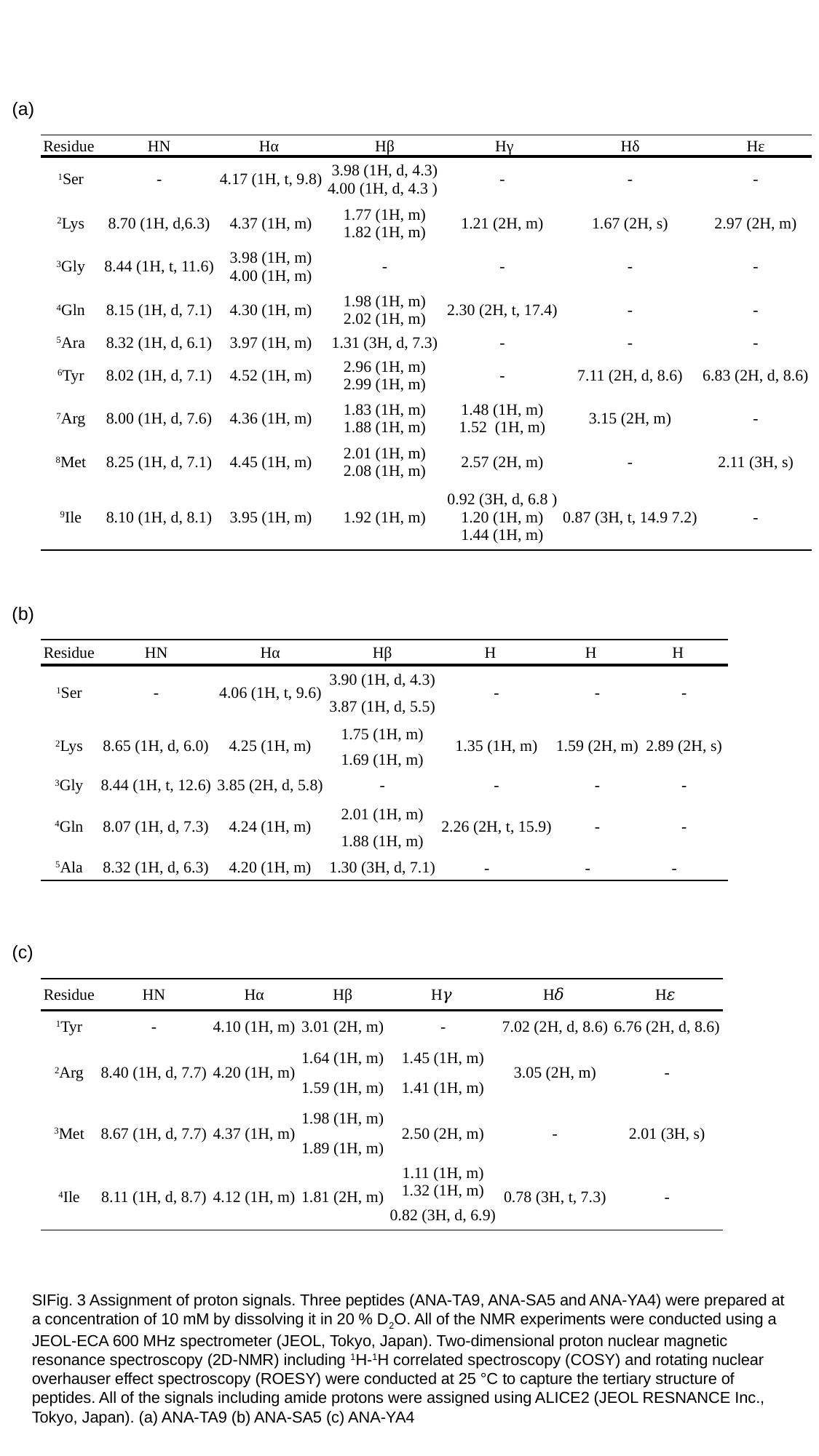

(a)
| Residue | HN | Hα | Hβ | Hγ | Hδ | Hε |
| --- | --- | --- | --- | --- | --- | --- |
| 1Ser | - | 4.17 (1H, t, 9.8) | 3.98 (1H, d, 4.3) 4.00 (1H, d, 4.3 ) | - | - | - |
| 2Lys | 8.70 (1H, d,6.3) | 4.37 (1H, m) | 1.77 (1H, m) 1.82 (1H, m) | 1.21 (2H, m) | 1.67 (2H, s) | 2.97 (2H, m) |
| 3Gly | 8.44 (1H, t, 11.6) | 3.98 (1H, m) 4.00 (1H, m) | - | - | - | - |
| 4Gln | 8.15 (1H, d, 7.1) | 4.30 (1H, m) | 1.98 (1H, m) 2.02 (1H, m) | 2.30 (2H, t, 17.4) | - | - |
| 5Ara | 8.32 (1H, d, 6.1) | 3.97 (1H, m) | 1.31 (3H, d, 7.3) | - | - | - |
| 6Tyr | 8.02 (1H, d, 7.1) | 4.52 (1H, m) | 2.96 (1H, m) 2.99 (1H, m) | - | 7.11 (2H, d, 8.6) | 6.83 (2H, d, 8.6) |
| 7Arg | 8.00 (1H, d, 7.6) | 4.36 (1H, m) | 1.83 (1H, m) 1.88 (1H, m) | 1.48 (1H, m) 1.52 (1H, m) | 3.15 (2H, m) | - |
| 8Met | 8.25 (1H, d, 7.1) | 4.45 (1H, m) | 2.01 (1H, m) 2.08 (1H, m) | 2.57 (2H, m) | - | 2.11 (3H, s) |
| 9Ile | 8.10 (1H, d, 8.1) | 3.95 (1H, m) | 1.92 (1H, m) | 0.92 (3H, d, 6.8 ) 1.20 (1H, m) 1.44 (1H, m) | 0.87 (3H, t, 14.9 7.2) | - |
(b)
| Residue | HN | Hα | Hβ | H𝛾 | H𝛿 | H𝜀 |
| --- | --- | --- | --- | --- | --- | --- |
| 1Ser | - | 4.06 (1H, t, 9.6) | 3.90 (1H, d, 4.3) | - | - | - |
| | | | 3.87 (1H, d, 5.5) | | | |
| 2Lys | 8.65 (1H, d, 6.0) | 4.25 (1H, m) | 1.75 (1H, m) | 1.35 (1H, m) | 1.59 (2H, m) | 2.89 (2H, s) |
| | | | 1.69 (1H, m) | | | |
| 3Gly | 8.44 (1H, t, 12.6) | 3.85 (2H, d, 5.8) | - | - | - | - |
| 4Gln | 8.07 (1H, d, 7.3) | 4.24 (1H, m) | 2.01 (1H, m) | 2.26 (2H, t, 15.9) | - | - |
| | | | 1.88 (1H, m) | | | |
| 5Ala | 8.32 (1H, d, 6.3) | 4.20 (1H, m) | 1.30 (3H, d, 7.1) | - | - | - |
(c)
| Residue | HN | Hα | Hβ | H𝛾 | H𝛿 | H𝜀 |
| --- | --- | --- | --- | --- | --- | --- |
| 1Tyr | - | 4.10 (1H, m) | 3.01 (2H, m) | - | 7.02 (2H, d, 8.6) | 6.76 (2H, d, 8.6) |
| 2Arg | 8.40 (1H, d, 7.7) | 4.20 (1H, m) | 1.64 (1H, m) | 1.45 (1H, m) | 3.05 (2H, m) | - |
| | | | 1.59 (1H, m) | 1.41 (1H, m) | | |
| 3Met | 8.67 (1H, d, 7.7) | 4.37 (1H, m) | 1.98 (1H, m) | 2.50 (2H, m) | - | 2.01 (3H, s) |
| | | | 1.89 (1H, m) | | | |
| 4Ile | 8.11 (1H, d, 8.7) | 4.12 (1H, m) | 1.81 (2H, m) | 1.11 (1H, m) 1.32 (1H, m) | 0.78 (3H, t, 7.3) | - |
| | | | | 0.82 (3H, d, 6.9) | | |
SIFig. 3 Assignment of proton signals. Three peptides (ANA-TA9, ANA-SA5 and ANA-YA4) were prepared at a concentration of 10 mM by dissolving it in 20 % D2O. All of the NMR experiments were conducted using a JEOL-ECA 600 MHz spectrometer (JEOL, Tokyo, Japan). Two-dimensional proton nuclear magnetic resonance spectroscopy (2D-NMR) including 1H-1H correlated spectroscopy (COSY) and rotating nuclear overhauser effect spectroscopy (ROESY) were conducted at 25 °C to capture the tertiary structure of peptides. All of the signals including amide protons were assigned using ALICE2 (JEOL RESNANCE Inc., Tokyo, Japan). (a) ANA-TA9 (b) ANA-SA5 (c) ANA-YA4

## Slide 4
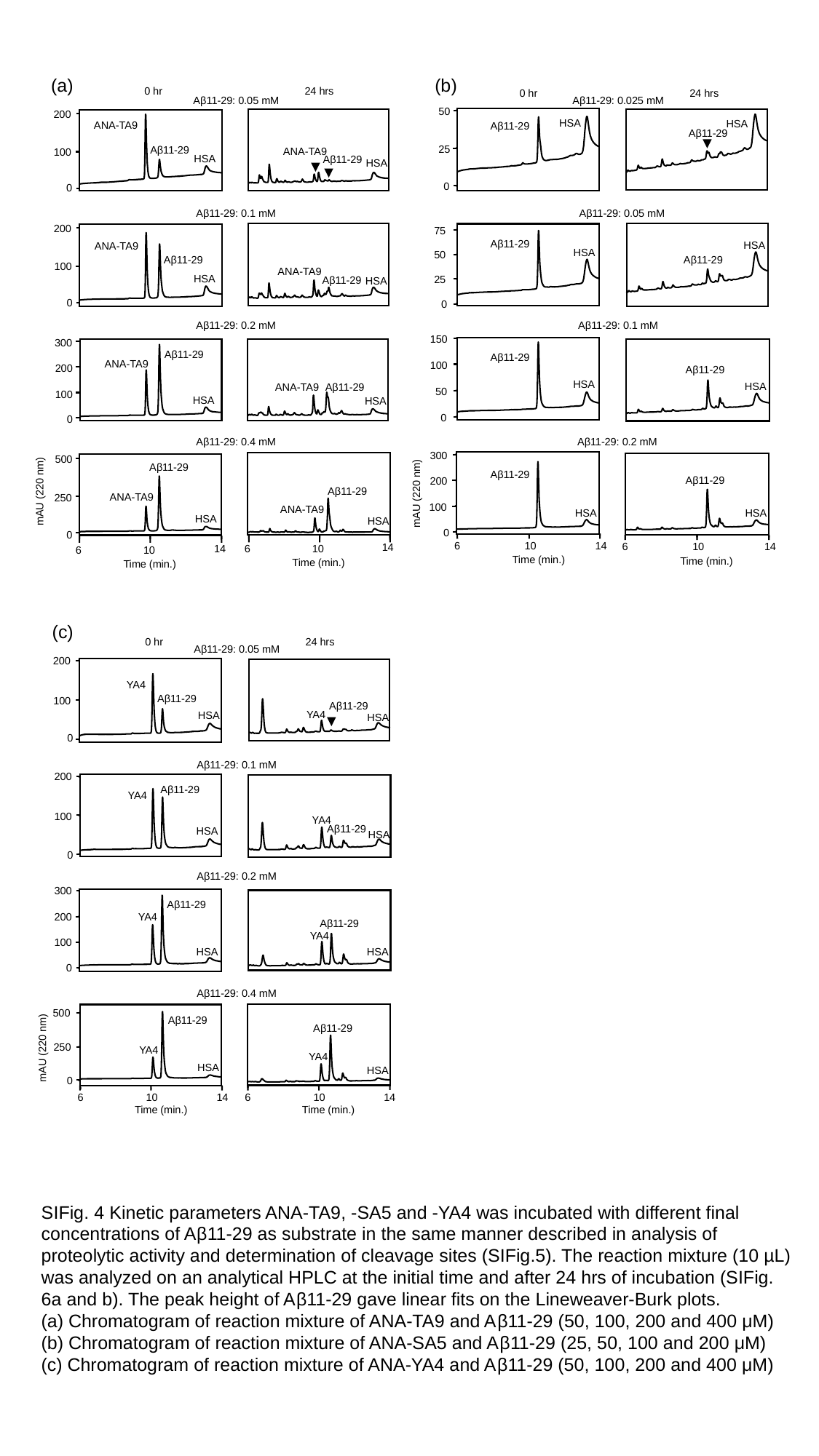

(a)
(b)
24 hrs
0 hr
0 hr
24 hrs
Aβ11-29: 0.05 mM
Aβ11-29: 0.025 mM
50
200
Aβ11-29
HSA
HSA
ANA-TA9
Aβ11-29
25
Aβ11-29
ANA-TA9
100
HSA
HSA
0
0
Aβ11-29: 0.1 mM
Aβ11-29: 0.05 mM
200
100
0
75
50
25
0
Aβ11-29
HSA
ANA-TA9
HSA
Aβ11-29
Aβ11-29
ANA-TA9
HSA
Aβ11-29
HSA
Aβ11-29: 0.2 mM
Aβ11-29: 0.1 mM
150
100
50
0
300
200
100
0
Aβ11-29
Aβ11-29
ANA-TA9
Aβ11-29
HSA
HSA
Aβ11-29
ANA-TA9
HSA
HSA
Aβ11-29: 0.4 mM
Aβ11-29: 0.2 mM
300
200
100
0
500
Aβ11-29
Aβ11-29
Aβ11-29
mAU (220 nm)
Aβ11-29
ANA-TA9
250
ANA-TA9
HSA
HSA
HSA
HSA
0
6
10
14
6
10
14
14
14
6
10
6
10
Time (min.)
Time (min.)
Time (min.)
Time (min.)
24 hrs
0 hr
Aβ11-29: 0.05 mM
200
100
0
YA4
Aβ11-29
Aβ11-29
YA4
HSA
HSA
Aβ11-29: 0.1 mM
200
100
0
Aβ11-29
YA4
YA4
Aβ11-29
HSA
HSA
Aβ11-29: 0.2 mM
300
200
100
0
Aβ11-29
YA4
Aβ11-29
YA4
HSA
HSA
Aβ11-29: 0.4 mM
500
250
0
Aβ11-29
Aβ11-29
YA4
mAU (220 nm)
YA4
HSA
HSA
6
10
14
6
10
14
Time (min.)
Time (min.)
Aβ11-29
mAU (220 nm)
(c)
SIFig. 4 Kinetic parameters ANA-TA9, -SA5 and -YA4 was incubated with different final concentrations of Aβ11-29 as substrate in the same manner described in analysis of proteolytic activity and determination of cleavage sites (SIFig.5). The reaction mixture (10 µL) was analyzed on an analytical HPLC at the initial time and after 24 hrs of incubation (SIFig. 6a and b). The peak height of Aβ11-29 gave linear fits on the Lineweaver-Burk plots.
(a) Chromatogram of reaction mixture of ANA-TA9 and Aβ11-29 (50, 100, 200 and 400 μM)
(b) Chromatogram of reaction mixture of ANA-SA5 and Aβ11-29 (25, 50, 100 and 200 μM)
(c) Chromatogram of reaction mixture of ANA-YA4 and Aβ11-29 (50, 100, 200 and 400 μM)

## Slide 5
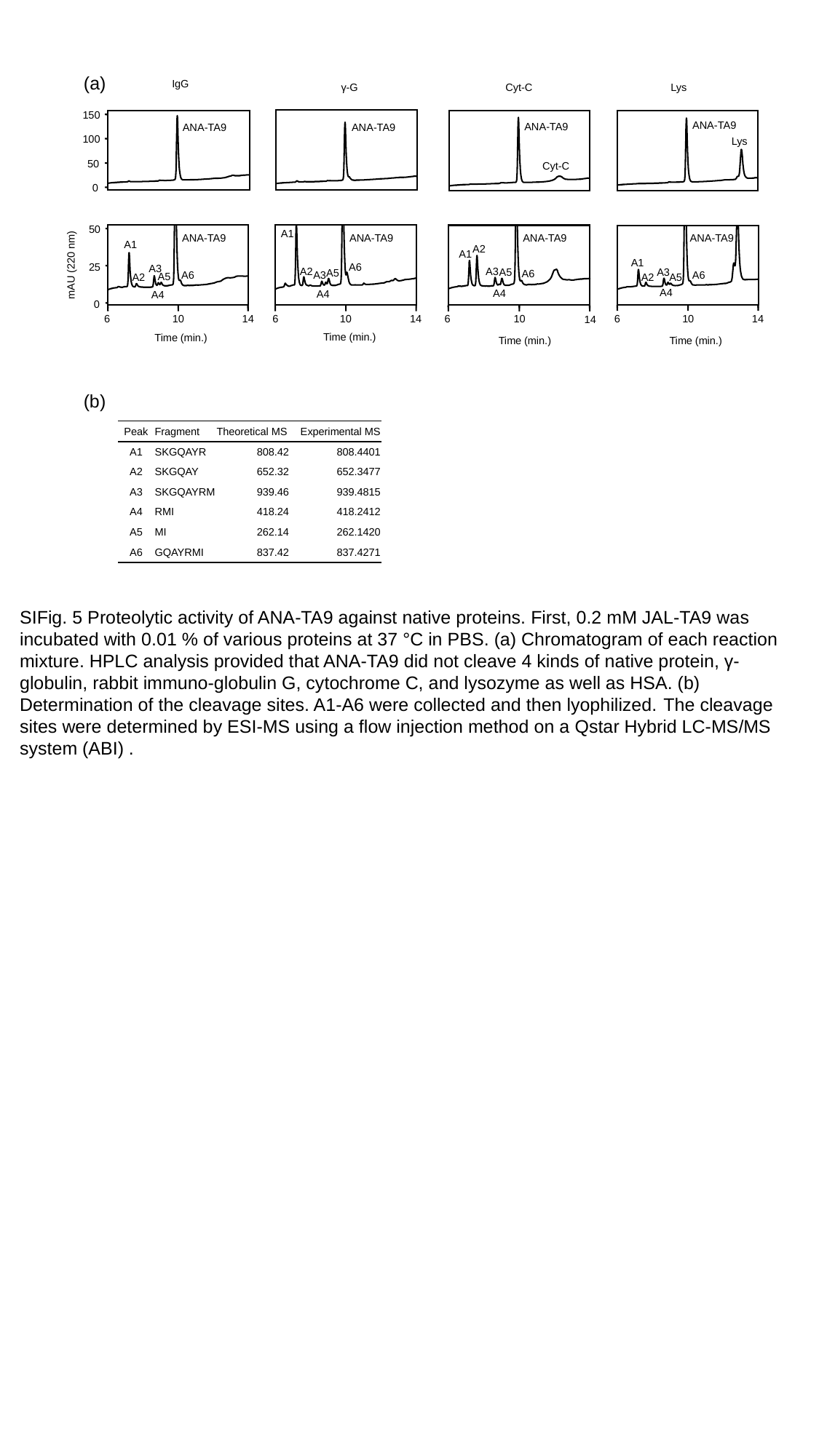

(a)
IgG
Lys
γ-G
Cyt-C
150
ANA-TA9
ANA-TA9
ANA-TA9
ANA-TA9
Lys
100
Cyt-C
50
0
A1
50
25
0
6
10
14
ANA-TA9
ANA-TA9
ANA-TA9
ANA-TA9
A1
A2
A1
A1
mAU (220 nm)
A6
A3
A3
A2
A3
A5
A5
A6
A6
A3
A6
A5
A5
A2
A2
A4
A4
A4
A4
6
10
6
10
14
6
10
14
14
Time (min.)
Time (min.)
Time (min.)
Time (min.)
(b)
| | | | |
| --- | --- | --- | --- |
| Peak | Fragment | Theoretical MS | Experimental MS |
| A1 | SKGQAYR | 808.42 | 808.4401 |
| A2 | SKGQAY | 652.32 | 652.3477 |
| A3 | SKGQAYRM | 939.46 | 939.4815 |
| A4 | RMI | 418.24 | 418.2412 |
| A5 | MI | 262.14 | 262.1420 |
| A6 | GQAYRMI | 837.42 | 837.4271 |
| | | | |
SIFig. 5 Proteolytic activity of ANA-TA9 against native proteins. First, 0.2 mM JAL-TA9 was incubated with 0.01 % of various proteins at 37 °C in PBS. (a) Chromatogram of each reaction mixture. HPLC analysis provided that ANA-TA9 did not cleave 4 kinds of native protein, γ-globulin, rabbit immuno-globulin G, cytochrome C, and lysozyme as well as HSA. (b) Determination of the cleavage sites. A1-A6 were collected and then lyophilized. The cleavage sites were determined by ESI-MS using a flow injection method on a Qstar Hybrid LC-MS/MS system (ABI) .

## Slide 6
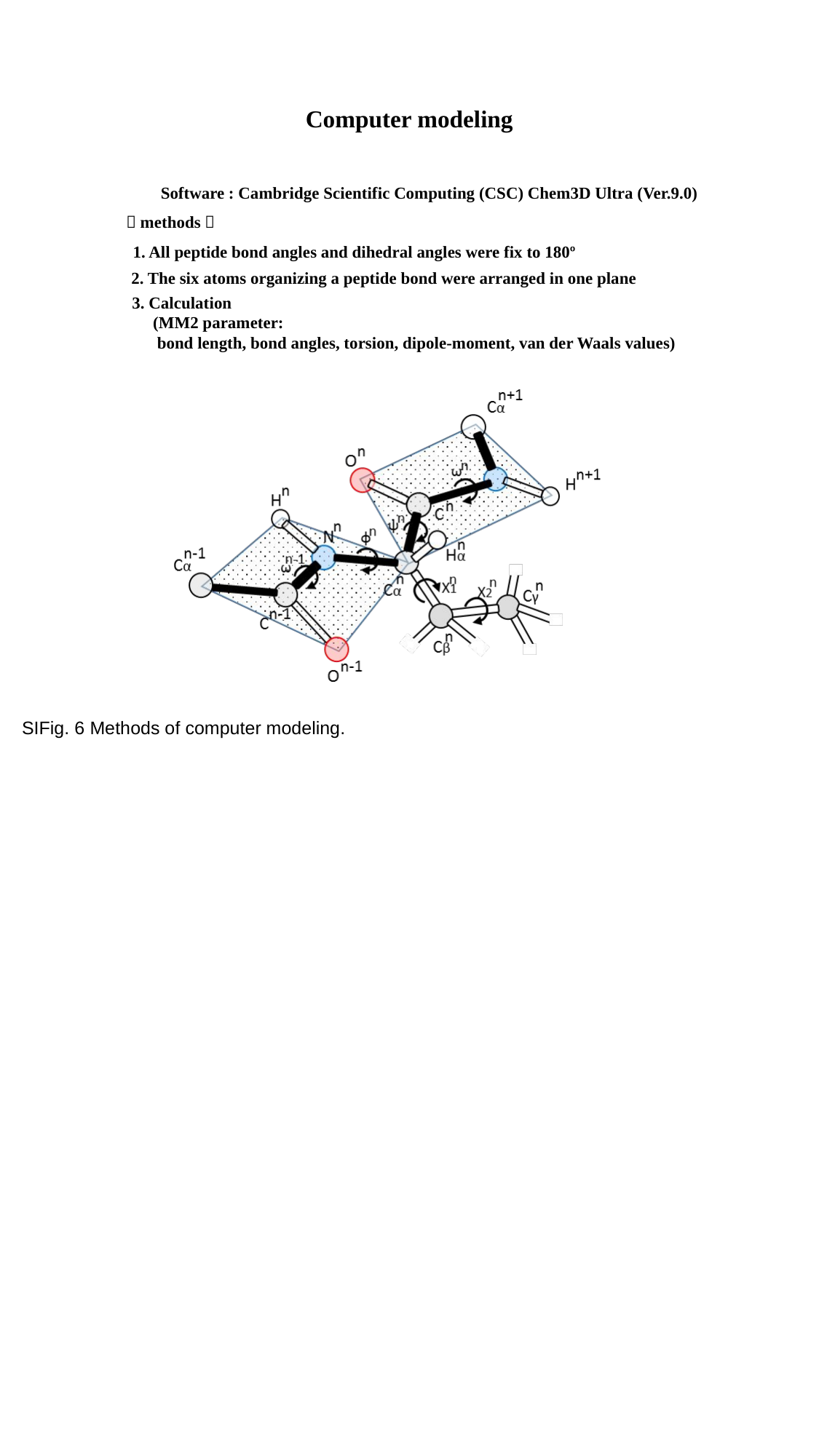

Computer modeling
Software : Cambridge Scientific Computing (CSC) Chem3D Ultra (Ver.9.0)
＜methods＞
1. All peptide bond angles and dihedral angles were fix to 180º
2. The six atoms organizing a peptide bond were arranged in one plane
3. Calculation
 (MM2 parameter:
 bond length, bond angles, torsion, dipole-moment, van der Waals values)
SIFig. 6 Methods of computer modeling.
